# Carotid atherosclerotic plaque microcalcification is independently associated with recurrent neurovascular events: A pilot study

**DOI:** 10.1177/17474930241264734

**Published:** 2024-07-27

**Authors:** Shiv Bhakta, Jason M Tarkin, Mohammed M Chowdhury, James HF Rudd, Elizabeth A Warburton, Nicholas R Evans

**Affiliations:** 1 Department of Clinical Neurosciences, University of Cambridge, Cambridge, UK; 2 Department of Medicine, University of Cambridge, Cambridge, UK; 3 Department of Vascular Surgery, University of Cambridge, Cambridge, UK

**Keywords:** Atherothrombotic, carotid plaque, carotid stenosis, radiology, vascular events, ischemic stroke

## Abstract

**Background:**

Microcalcification and macrocalcification are critical processes in atherosclerotic plaque progression, though how these processes relate to the risk of stroke recurrence in symptomatic carotid atherosclerosis is poorly understood.

**Methods:**

We performed a post hoc analysis of data from the ICARUSS (Imaging Carotid Atherosclerosis in the Recovery and Understanding of Stroke Severity) study, where individuals with acute ischemic stroke originating from ipsilateral carotid stenosis of ⩾ 50% underwent ^18^F-sodium fluoride positron emission tomography (NaF-PET) to measure microcalcification. Tracer uptake was quantified using maximum tissue-to-background ratio (TBR_max_). Macrocalcification was measured on computed tomography (CT) using Agatston scoring. Patients were followed up for 6 months for recurrent ipsilateral neurovascular events.

**Results:**

Five (27.8%) of 18 individuals had a recurrent ischemic stroke or transient ischemic attack. Ipsilateral carotid plaque NaF uptake at baseline was higher in those with recurrent events compared to those without, and this association remained after adjustment for other vascular risk factors (adjusted odds ratio (aOR) = 1.24, 1.03–1.50). Macrocalcification score in the symptomatic artery was also significantly independently associated with ipsilateral recurrence, but the effect size was relatively smaller (aOR = 1.12, 1.06–1.17 for each 100 unit increase).

**Conclusions:**

Our findings indicate that microcalcification in symptomatic carotid plaques is independently associated with ipsilateral ischemic stroke recurrence. Furthermore, differences in the extent of active microcalcification in macrocalcified plaques may help explain variation in the relationship between calcified carotid plaques and stroke recurrence reported in the literature. Our pilot study indicates that evaluation of carotid artery microcalcification using NaF-PET may be a useful method for risk-stratification of carotid atherosclerosis, though our findings require confirmation in larger cohorts.

## Introduction

Microcalcification and macrocalcification are related but distinct processes^1^ that represent different stages of atherogenesis.^2^ Microcalcification is a focal process, associated with local inflammation and plaque rupture,^3^ which can be identified *in vivo* using sodium fluoride positron emission tomography (NaF-PET).^4^ Prior studies have demonstrated increased NaF uptake in symptomatic carotid atheroma.^5,6^ However, to date, there has been no assessment of how NaF uptake relates to recurrent neurovascular events.

Measurement of coronary artery macrocalcification is an important clinical risk-stratification tool, and an independent predictor of cardiovascular events,^7^ performed in routine cardiology practice using non-contrast computed tomography (CT). However, the relationship between carotid artery calcium score (CACS) and stroke risk remains unclear. Some studies suggest the presence of macrocalcification is protective,^8,9^ while others suggest carotid macrocalcification is a marker of stroke risk.^10^

Our study investigates how NaF uptake and CACS in symptomatic carotid atherosclerosis relate to recurrent ipsilateral neurovascular events.

## Methods

### Study eligibility and recruitment

We describe post hoc analysis of data obtained from the ICARUSS (Imaging Carotid Atherosclerosis in the Recovery and Understanding of Stroke Severity) study,^6^ which prospectively recruited consecutive individuals presenting with first-ever ischemic stroke (confirmed on magnetic resonance imaging (MRI)) within the previous 7 days with ipsilateral common or internal carotid artery stenosis of ⩾ 50% on CT angiography or Doppler. The 50% threshold in the initial study was chosen to increase the likelihood that the carotid plaque was a culprit plaque, as well as increasing the clinical relevance of the study given this is the threshold at which surgical management is considered. A seven-day threshold was used as previous PET studies using fluorodeoxyglucose (FDG) indicated tracer signal decreased with time from the index event, and it was unknown if a similar pattern occurred with NaF. Individuals with atrial fibrillation and other confirmed non-large artery atherosclerosis stroke etiologies were also excluded from the ICARUSS study,^6^ in order to select those individuals with the highest likelihood that their stroke was secondary to the ipsilateral carotid plaque. In addition, all participants had MRI-confirmed infarcts that appeared embolic in pattern.

Seven (26.9%) patients in the ICARUSS study underwent carotid endarterectomy. The patients included in this sub-study of the ICARUSS dataset^6^ did not undergo endarterectomy, with factors influencing the decision not to operate including patient frailty and comorbidities, size of infarct, and patient wishes. Of the participants recruited to the ICARUSS study, all participants were reviewed for potential revascularization via multidisciplinary discussion based on the NASCET criteria, blinded to all research imaging results. All participants provided written informed consent. The medical management in these participants was determined by the attending physician, blinded to all research imaging results. This included best medical therapy of antiplatelet treatment with clopidogrel, along with high-dose atorvastatin, unless contraindicated.

The study was approved by a national research ethics committee (Nottingham One Research Ethics Committee, 14/EM/0128).

### PET/CT imaging

NaF-PET/CT imaging was performed using a GE Discovery 690 (GE Medical Systems Ltd, Hatfield, UK) with 64-slice CT within 14 days of ischemic stroke. Participants were injected intravenously with a target dose of 125 MBq of NaF followed by a 60-min uptake time.^11^ A CT carotid angiogram was performed concurrently.

### Radiotracer uptake quantification

Co-registered PET/CT images were resampled to 3 mm slice thickness and regions of interest (ROIs) drawn manually along the common and internal carotid artery to encompass the region 0.9 cm proximal and 3 cm distal to the carotid bifurcation.^12^ Tracer uptake within ROIs was measured using maximum standardized uptake values (SUV_max_). To correct for blood pool activity, arterial SUV_max_ was adjusted for venous SUV to give the maximum target-to-background ratio (TBR_max_). The 3 mm slice thickness was chosen to match the spatial resolution of PET (which is around 3 mm), as per standard technique in vascular PET imaging studies. Slice thicknesses below this would be subject to partial volume effects from the spatial resolution of PET.

TBR_max_ was measured in the most diseased segment (MDS) of the plaque—the most diseased 9 mm (three ROIs) of the artery, where the central ROI had the highest uptake in the artery.^13^—and across the whole vessel (WV)—the median tracer uptake for all 14 axial slices along the artery.^6^

PET imaging datasets were analyzed using OsiriX (version 5.7.1, OsiriX Imaging Software, Geneva, Switzerland). The primary reader performed readings independently, with an experienced reader (N.R.E.) providing reproducibility. Both readers were blinded to clinical information and symptomatic artery.

### Macrocalcification scoring

Macrocalcification was scored as a product of weighted density and area.^14^ CACS were measured from unenhanced CT carotid angiograms using the Calcium Scoring Plugin (version 1.0) in OsiriX. The detection threshold for calcification was set at 130 HU (Hounsfield unit).

### Clinical outcomes

Participants were followed up for 6 months and underwent assessment for recurrent ipsilateral neurovascular events by an experienced stroke clinician (N.R.E./E.A.W.), blinded to PET readings. Patients with recurrent events were treated by their attending physician blinded to all research imaging data. Their clinical workup included assessment of the cause of the recurrent event.

### Statistical analysis

The Shapiro–Wilk test was used to assess for normality of continuous data. Parametric data were reported as mean ± SD and non-parametric data reported as median and interquartile range (IQR). In unpaired groups, parametric and non-parametric data were compared using *t*-testing or Wilcoxon rank-sum testing, respectively. Correlations were tested using two-tailed Spearman ρ correlation (nonparametric or ordinal data) or Pearson correlation coefficient (parametric data). The cut-off for statistical significance was set at 5%. Inter-rater reproducibility was calculated using intra-class correlation coefficients (ICC).^11^ Multivariable logistic regression for ipsilateral recurrence included any variable on univariable analysis that had a significance below p = 0.10. Data were analyzed using R (version 4.2.2, 2022, R Foundation for Statistical Computing, Vienna, Austria). This post hoc analysis is of data obtained from Evans et al,^6^ with that study not powered to identify any impact on recurrence related to microcalcification signal on PET.

## Results

### Study population

Twenty-seven individuals underwent NaF-PET/CT. Imaging was uninterpretable in one participant, seven participants underwent carotid endarterectomy (and were excluded), and one participant died of a non-vascular cause within the 6-month follow-up period. Therefore, data from 18 participants were analyzed for potential associations between micro/macrocalcification using NaF-PET signal or CACS, and stroke recurrence. Five (27.8%) of the included participants had clinically confirmed recurrent ipsilateral neurovascular events within the follow-up period. The clinical characteristics of the included participants is shown in Table 1. In those with recurrence, in no instances were there subsequent detection of an alternative etiology (in particular, no atrial fibrillation was detected) or plaque progression. No individuals were found to have intracranial artery disease or aortic plaques. Of those with recurrent ipsilateral events, the carotid artery risk (CAR) score,^15^ was significantly higher than in those with no recurrent events (median 21 (IQR = 5) vs 8 (IQR = 4.5), p = 0.009). Of note, there was no significant difference in the CAR score between those participants who underwent carotid endarterectomy, and those who did not have surgical intervention (median 9 (IQR = 4.5) vs 10 (IQR = 8.75), p = 0.86). Table 1 also includes demographic data for those ICARUSS participants who underwent carotid endarterectomy, with p-values given comparing the revascularized versus the non-revascularized participants in the original ICARUSS study.

**Table 1.** Clinical characteristics of study cohort.

	Recurrence (n = 5)	No recurrence (n = 13)	p-value	ICARUSS CEA (n = 7)	p-value
Mean age (years)	84.0 (SD 8.28)	72.7 (SD 9.78)	0.04	74.0 (SD 6.86)	0.62
Men (n)	4 (80.0%)	8 (61.5%)	0.50	5 (71.4%)	0.85
Median BMI (kg/m^2^)	27.8 (IQR 7.1)	26.5 (IQR 6.7)	0.84	25.6 (IQR 2.2)	0.72
Smoking history (n)	3 (60.0%)	9 (69.2%)	0.76	4 (57.1%)	0.69
Diabetes mellitus (n)	1 (20.0%)	2 (15.4%)	0.88	1 (14.3%)	0.92
Hypertension (n)	5 (100%)	9 (69.2%)	0.19	3 (42.9%)	0.11
Statin before index event (n)	3 (60.0%)	5 (38.5%)	0.46	1 (14.3%)	0.18
Antiplatelet before index event (n)	4 (80.0%)	4 (30.8%)	0.08	0 (0%)	0.04
Cardiovascular history (n)	3 (60.0%)	4 (30.8%)	0.29	1 (14.3%)	0.26
Median NIHSS	6 (IQR 10)	4 (IQR 11)	0.22	4 (IQR 6)	0.54
Thrombolyzed (n)	0 (0%)	2 (15.4%)	0.42	4 (57.1%)	0.02
Modal degree of symptomatic stenosis	70–89%	50–69%/> 90%		> 90%	
Modal degree of asymptomatic stenosis	1–29%/30–49%	30–49%		1–29%	

ICARUSS: Imaging Carotid Atherosclerosis in the Recovery and Understanding of Stroke Severity; CEA: carotid endarterectomy; BMI: body mass index; IQR: interquartile range; NIHSS: National Institute of Health Stroke Scale score.

Smoking history indicates current or ex-smokers; cardiovascular history indicates those with previous ischemic heart disease or myocardial infarction. The cohort who underwent CEA were compared to the pooled cohort of the two groups in this study (recurrence and no recurrence) who did not undergo CEA.

### Culprit plaque NaF uptake and recurrence

**Figure 1.**
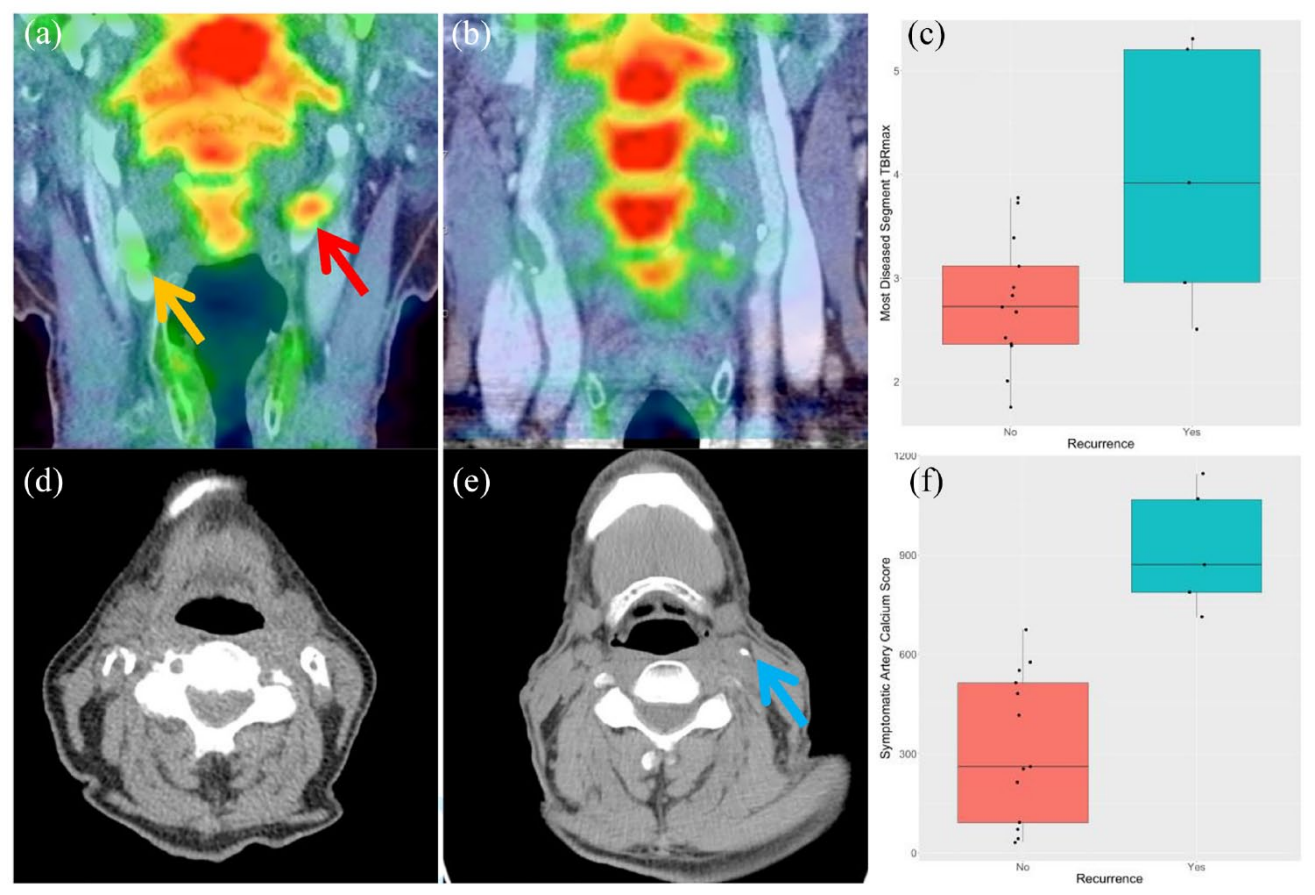
NaF uptake, Agatston calcium scores, and their relationship with stroke recurrence. (a) Coronal PET/CT demonstrating high NaF uptake (culprit artery—red arrow, non-culprit artery—gold arrow). (b) Coronal PET/CT demonstrating low NaF uptake in the carotids. (c) Boxplot comparing most diseased segment (MDS) maximum TBR (TBR_max_) in those with recurrent stroke versus those without. (d) Axial noncontrast-enhanced CT demonstrating a high calcium burden in the carotid artery (white arrows). (e) Axial non-contrast-enhanced CT demonstrating a low calcium burden in the carotid artery (blue arrow). (f) A boxplot comparing symptomatic carotid artery calcium scores in those having a recurrent stroke versus those without recurrence.

Median NaF MDS TBR_max_ was higher in individuals with recurrent ipsilateral neurovascular events (3.92 (2.96–5.20) vs 2.72 (2.36–3.12) p = 0.04). However, median WV TBR_max_ was not significantly higher in those with recurrent events (2.50 (2.35–2.55) vs 2.01 (1.86–2.13) p = 0.11; see Figure 1). This relationship between NaF MDS TBR_max_ and recurrent events remained after correction for age and prior antiplatelet use (see Table 2). The inter-rater ICC was 0.94.

**Table 2.** Logistic regression model analysis of recurrent ipsilateral neurovascular events according to most diseased segment NaF TBR_max_.

	Crude model		Model 1		Model 2	
	OR	95% CI	aOR	95% CI	aOR	95% CI
MDS TBR_max_	1.31	1.08–1.58	1.24	1.03–1.50	1.22	1.01–1.48

NaF: sodium fluoride; MDS: most diseased segment; TBR_max_: maximum tissue-to-background ratio; OR: odds ratio; CI: confidence interval; aOR: adjusted odds ratio.

The crude model is the unadjusted analysis, while Model 1 adjusts for age, and Model 2 adjusts for age and antiplatelet use prior to the index event in the ICARUSS (Imaging Carotid Atherosclerosis in the Recovery and Understanding of Stroke Severity) study (both p < 0.10 between the recurrence and non-recurrence groups as per Table 1).

### Macrocalcification and recurrence

On univariable analysis, CACS in the symptomatic carotid was significantly higher for those with ipsilateral recurrent neurovascular events (median 871 (787–1067) vs 261 (92–513) p = 0.0002) than those without. Total carotid calcium burden (the score of both ipsilateral and contralateral carotid arteries) was also higher in those with recurrent neurovascular events (1358 (1343–1658) vs 615 (394–708) p = 0.002). A significant relationship between both symptomatic artery and total CACS and recurrent events remained after correction for age and prior antiplatelet use (see Table 3).

**Table 3.** Logistic regression model analysis of recurrent ipsilateral neurovascular events according to symptomatic carotid artery and total carotid artery calcium score (CACS).

	Crude model		Model 1		Model 2	
	OR	95% CI	aOR	95% CI	aOR	95% CI
Symptomatic CACS (per 100 unit increase)	1.11	1.07–1.15	1.12	1.06–1.17	1.11	1.05–1.17
Total CACS (per 100 unit increase)	1.06	1.04–1.09	1.08	1.03–1.11	1.07	1.03–1.11

OR: odds ratio; CI: confidence interval; aOR: adjusted odds ratio.

The crude model is the unadjusted analysis, while Model 1 adjusts for age, and Model 2 adjusts for age and antiplatelet use prior to the index event in the ICARUSS (Imaging Carotid Atherosclerosis in the Recovery and Understanding of Stroke Severity) study (both p < 0.10 between the recurrence and non-recurrence groups as per Table 1).

The inter-rater ICC for CACS was 0.96. There was a moderate correlation between symptomatic artery CACS and MDS TBR_max_ (r_s_ = 0.49, p = 0.04), and between total CACS and MDS TBR_max_ (r_s_ = 0.58, p = 0.01).

## Discussion

Our results indicate that higher NaF uptake—and the microcalcification it represents—is independently associated with ipsilateral stroke recurrence in symptomatic carotid atherosclerosis. In keeping with previous results,^6^ we found it was the focal uptake of NaF in the plaque at the MDS of atherosclerosis—rather than uptake along the length of the whole artery—that was associated with future events.

We also found that symptomatic carotid atheroma with higher calcium scores, as well as a higher global burden of macrocalcification, were associated with ipsilateral stroke recurrence, though the effect size appeared smaller relative to that seen with microcalcification. Carotid macrocalcification reflects vascular risk factors,^16,17^ and hence stroke risk, but the finding that ipsilateral recurrence was significantly associated with symptomatic carotid plaque CACS suggests that the nature of the plaque itself may contribute to recurrent thromboembolic risk.

The pattern of these results indicates that plaques with “active microcalcification” (i.e. those identified by higher NaF uptake) are more likely to contribute to ongoing plaque disruption and subsequent recurrent thromboemboli, moreso than the burden of macrocalcification. The difference in macrocalcific plaques with different levels of active microcalcification—effectively stable versus unstable macrocalcific plaques—may account for the inconsistent findings found in the literature between carotid macrocalcification and stroke recurrence.

Related to this, previous vascular NaF-PET studies have shown varying associations between NaF signal and macrocalcification.^18,19^ Although our results found an association between NaF and calcium score, the reported presence of distinct regions of microcalcification and macrocalcification indicates these are separate—though related—processes, again consistent with concept that macrocalcific plaques may vary in terms of active microcalcific activity and stability.

This study did not include assessment of other plaque characteristics. Furthermore, the sample size of this proof-of-principle study, as well as the small number of recurrent events, limits multifactorial analysis, and so our findings should be interpreted as hypothesis-generating. Validation in a larger cohort or meta-analysis will be important. However, the novel finding of the role of microcalcification, which cannot be detected by MRI, adds an additional factor to consider when assessing plaque vulnerability. These limitations represent important avenues for future work, including through the use of hybrid PET/MRI imaging,^20^ which could facilitate better understanding of how the microcalcification noted on PET imaging relates to other plaque morphological characteristics described on MRI.

Our study cohort—individuals with significant symptomatic carotid stenosis but not undergoing endarterectomy—represent a selected group of individuals, but one that is encountered commonly in clinical practice and for whom improved prognostication may be most beneficial. A variety of factors beyond the degree of stenosis influence the decision to operate^21^—including pre-morbid frailty, extent of disability, time since event, and patient wishes—and in such borderline cases where benefits and risks are felt to be finely balanced, better personalized risk-stratification may help inform clinical management decisions to improve outcomes. Whether our findings provide the mechanistic underpinnings or additional information for risk stratification relating the higher CAR score in the cohort with recurrence would also be a valuable topic for future work.

Unlike for PET scans using the most commonly used radiotracer FDG, individuals undergoing NaF-PET are not required to fast prior to imaging. There are also other favorable factors with NaF-PET, including a shorter tracer uptake time (60 min) and minimal blood-pooling. Although typically well-tolerated by patients, availability and capacity of PET facilities, the additional radiation exposure (2.2 mSv per administration), and cost may have implications for the use of NaF-PET in routine clinical practice. Potential future clinical applications of carotid PET imaging may therefore be best prioritized to those with uncertain or borderline risk as assessed by conventional clinical methods.

These pilot study findings may have the potential to inform future strategies to evaluate the risk of recurrent events in carotid atherosclerosis. Furthermore, the role of microcalcification in recurrent stroke events may represent a novel therapeutic target for plaque stabilization. These proof-of principle findings require validation in larger studies/meta-analyses.

## Conclusion

This is the first study to show the association between NaF uptake in carotid atherosclerosis and ipsilateral stroke recurrence. Our data also suggest a link between the burden of macrocalcification and recurrent ipsilateral events. These findings need to be replicated in larger studies, but have potential implications for our understanding of carotid atherosclerosis and its clinical management.
